# Human Neural Stem Cell Transplantation Rescues Cognitive Defects in APP/PS1 Model of Alzheimer’s Disease by Enhancing Neuronal Connectivity and Metabolic Activity

**DOI:** 10.3389/fnagi.2016.00282

**Published:** 2016-11-23

**Authors:** Xueyuan Li, Hua Zhu, Xicai Sun, Fuxing Zuo, Jianfeng Lei, Zhanjing Wang, Xinjie Bao, Renzhi Wang

**Affiliations:** ^1^Department of Neurosurgery, Peking Union Medical College Hospital, Chinese Academy of Medical Sciences and Peking Union Medical CollegeBeijing, China; ^2^Department of Pathology, Comparative Medical Center, Peking Union Medical College and Institute of Laboratory Animal Science, Chinese Academy of Medical ScienceBeijing, China; ^3^Center for Stem Cell Biology and Regenerative Medicine, Center for Life Sciences, School of Medicine, Tsinghua UniversityBeijing, China; ^4^Center for Medical Experiments and Testing, Capital Medical UniversityBeijing, China

**Keywords:** APP/PS1 mice, neuronal connectivity, metabolic activity, cognition, human neural stem cell, Alzheimer’s disease

## Abstract

Alzheimer’s disease (AD), the most frequent type of dementia, is featured by Aβ pathology, neural degeneration and cognitive decline. To date, there is no cure for this disease. Neural stem cell (NSC) transplantation provides new promise for treating AD. Many studies report that intra-hippocampal transplantation of murine NSCs improved cognition in rodents with AD by alleviating neurodegeneration via neuronal complement or replacement. However, few reports examined the potential of human NSC transplantation for AD. In this study, we implanted human brain-derived NSCs (hNSCs) into bilateral hippocampus of an amyloid precursor protein (APP)/presenilin 1 (PS1) transgenic (Tg) mouse model of AD to test the effects of hNSC transplantation on Alzheimer’s behavior and neuropathology. Six weeks later, transplanted hNSCs engrafted into the brains of AD mice, migrated dispersedly in broad brain regions, and some of them differentiated into neural cell types of central nervous system (CNS). The hNSC transplantation restored the recognition, learning and memory deficits but not anxiety tasks in AD mice. Although Aβ plaques were not significantly reduced, the neuronal, synaptic and nerve fiber density was significantly increased in the frontal cortex and hippocampus of hNSC-treated AD mice, suggesting of improved neuronal connectivity in AD brains after hNSC transplantation. Ultrastructural analysis confirmed that synapses and nerve fibers maintained relatively well-structured shapes in these mice. Furthermore, *in vivo* magnetic resonance spectroscopy (MRS) showed that hNSC-treated mice had notably increased levels of N-acetylaspartate (NAA) and Glu in the frontal cortex and hippocampus, suggesting that neuronal metabolic activity was improved in AD brains after hNSC transplantation. These results suggest that transplanted hNSCs rescued Alzheimer’s cognition by enhancing neuronal connectivity and metabolic activity through a compensation mechanism in APP/PS1 mice. This study provides preclinical evidence that hNSC transplantation can be a possible and feasible strategy for treating patients with AD.

## Introduction

In 2015 Alzheimer’s report shows that 46 million people are now suffering from Alzheimer’s disease (AD) worldwide currently, causing trillions of dollars for care each year (Prince et al., 2015). Featured by Aβ formation and neuronal loss in broad brain regions, AD is associated with progressive decline of cognitive functions and ultimately ends in death (Li et al., 2015). Despite much progress in the pathogenesis, no curative measures have been developed to improve the poor prognosis of patients with AD. Currently, the widely-used therapies including cholinesterase inhibitors and N-methyl D-aspartic acid (NMDA) receptor antagonists are symptomatic (Fereshtehnejad et al., 2014), which have diminished efficacy with long-term medication and always bring considerable side effects (Zhang N. et al., 2014).

With advances of stem cell techniques, using neural stem cells (NSCs) to repair or complement damaged neurons has become an appealing strategy for treating AD (Li et al., 2015). Since NSCs have the ability of self-renew and multi-cellular differentiation and are endowed with destination to neural cells, they are ideal sources for cell therapies for AD. In recent ages, a number of studies have reported the transplantation of NSCs in animal studies of AD (Blurton-Jones et al., 2009; Chen et al., 2012; Kim et al., 2015). It has been shown that transplanted NSCs could survive and differentiate in the brain of AD mice with cognitive or memory improvement. However, the majority of studies are limited to animal experiments, and the sources of NSCs are majorly derived from the same species as recipients. In rare studies, the potential of human-derived NSCs (hNSCs) has been evaluated for the treatment of AD. The absence of human involvement in animal studies makes it difficult to extrapolate experimental findings to clinical trials.

Furthermore, the evaluation of NSCs on AD in recent studies is limited to behavioral and histological observation. Few studies have assessed the changes of *in vivo* neurometabolites after NSC transplantation in AD brain (Shihabuddin and Aubert, 2010). Magnetic resonance spectroscopy (MRS) provides a *non-invasive* measure to quantitate brain metabolism and it is becoming widely used in researches of AD (Chen et al., 2012). The metabolites characteristic of AD include N-acetylaspartate (NAA), choline (Cho), glutamate (Glu), myo-inositol (mI) and creatine (Cr; Ackl et al., 2005; Zhang N. et al., 2014). While NAA and Glu were found to decrease in AD, mI exhibited an early increase (Zhang N. et al., 2014). Thus, MRS offers a sensitive and reliable tool to detect brain metabolic changes, which can be used to assess the effects of NSC transplantation on AD (Arturo et al., 2004).

In this study, we implanted hNSCs into bilateral hippocampus of amyloid precursor protein (APP)/presenilin 1 (PS1) transgenic (Tg) mice to assess the effects of hNSC transplantation on Alzheimer’s behavior and pathology. Because NSCs do not express mature antigen, we suppose that implanted hNSCs could survive and differentiate in mice brains. Simultaneously, we employed ^1^H-MRS to detect metabolic changes after NSC transplantation in AD mice brains. If implanted hNSCs can improve the neurological function and brain metabolism of AD mice, they are more likely to exert neuroprotective effects in human-self. The ultimate goal of this study is to provide preclinical evidence for using NSC therapies in the management of AD patients.

## Materials and Methods

### Animal

All animal experiments were approved by the Institutional Animal Care and Use Committee of the Institute of Laboratory Animal Science of Peking Union Medical College (ILAS-PL-2014-003). Animals were provided by the Institute of Experimental Animals of the Chinese Academy of Medical Science and provided with care according to the guidelines published in the National Institutes of Health Guide for Care and Use of Laboratory Animals.

Eight-month-old female APP/PS1 Tg mice and wild-type (WT) littermates were used in this study. All Tg mice expressed the Swedish (K670N/M671L) mutation of human APP together with PS1 deleted in exon nine on a C57BL/6J background, confirmed by PCR genotyping of mouse tail tissue. Twelve Tg mice were employed to receive hNSC transplantation (NSC group), 12 Tg mice were subject to PBS transplantation (PBS group) and 12 WT mice without any treatment were used as controls (WT group). All animals had free access to food and water and were housed in cages in an environmentally controlled room with a 12-h light/dark cycle. All procedures involving animals were performed according to guidelines published in the National Institutes of Health Guide for Care and Use of Laboratory Animals.

### Preparations of hNSCs

Human NSCs were obtained from a NSC line derived from human fetal brain (Angecon Biotech, Shanghai, China), transduced with the lentivirus-mediated gene encoding green fluorescent protein (GFP). Using the procedures as we reported previously (Zuo et al., 2015), primary single cell suspensions were isolated from human cortex tissues of legally terminated embryos under the supervision of National Health and Family Planning Commission of the People’s Republic of China. NSCs were cultured in the serum-free NSC medium (Angecon Biotech, Shanghai, China) at 37°C, 5% CO_2_, and 80% relative humidity, and amplified with a 7–10 day interval to the third generation. Neurosphere cultures were digested with 0.05% trypsin-EDTA (Invitrogen, Carlsbad, CA, USA) followed by trypsin inhibitor (Roche, Mannheim, Baden-Württemberg, Germany). Before transplantation, NSCs were stained for Nestin to confirm the nature of NSCs and then adjusted to a concentration of 10^5^/μl. All procedures were approved by Human Experimentation and Ethics Committee.

### Transplantation of hNSCs

Tg mice were anesthetized with intraperitoneal injection of 5% chloral hydrate (0.1 ml/10 g) and then fixed on a stereotaxic apparatus. The skin was shaved and sterilized with iodine complex. The scalp was dissected in the middle line and retracted bilaterally to expose the sagittal and coronal sutures. Based on Mouse Brain Stereotaxic Atlas, bilateral hippocampal CA1 regions were selected as sites for transplantation. Two vertical bone holes were drilled symmetrically at 1.8 mm besides sagittal suture and 2.0 mm posterior to anterior fontanel with anterior fontanel as the center. A Hamilton microsyringe fixed on the stereotaxic apparatus was inserted 2.5 mm under the dura, and a 4 μl NSC suspension (at 1 × 10^5^/μl) or PBS was injected into the brain gently (>10 min). The needle was maintained in place for 5 min and then was withdrawn at a slow pace (>3 min). After repeating washes with normal saline, the wound was sewed up and covered with sterile dressings.

### Behavioral Assessment

Behavioral assessment was performed as previously described (Zhang L. et al., 2014). The anxiety of mice was assessed using the open field test (OFT). In the test, each mouse was allowed to freely move in the open-field square apparatus (50 cm × 50 cm) divided into the peripheral and central zones. The movements of mice were monitored using a computer-driven movement tracing system (Ethovision, Noldus Information Technology, Wageningen, Netherlands). The time of mouse staying in both zones, as well as the frequency of mouse’s immobility, high mobility or immobility, were recorded. The high mobility and immobility were defined as mobility rates >60% and <20%, whereas the intermediate mobility was between 60% and 20%. After the OFT, novel object recognition (NOR) task was conducted to assess the recognition ability. In the task, two identical objects were positioned in the apparatus and the mice were allowed to explore in the field 5 min each day for three consecutive days. Twenty four hours later, one object was displaced with a novel object of different shape and color, and again mice explored the field. Each score was recorded for the mouse when its nose touched the object or its head was oriented towards the object within a 1-cm distance. The exploration time for the familiar (*T*_F_) or novel object (*T*_N_) during the test was collected and the recognition ability was calculated using the discrimination index (DI; Yao et al., 2013; Zhang L. et al., 2014) as the following formula: DI = (*T*_N_ − *T*_F_)/(*T*_N_ + *T*_F_) × 100%.

The spatial memory learning and memory was assessed using Morris water maze (MWM) test. The test was conducted in a white circular pool (100-cm diameter) divided into four equal quadrants. The pool was filled with water made opaque with non-fat milk at a temperature of 22 ± 1°C. First, mice were allowed to swim freely for 60 s in the pool with a visible platform. Then, an escape platform (6-cm diameter) was placed in the center of quadrant II, submerged 1 cm below the water surface. Each mouse was given three trials each lasting 60 s for five successive days, and in each trial the mice were allowed to swim freely to escape on the platform. The time taken to the platform (escape latency) was recorded and the values of three trials were averaged. At 24 h after the last set of trials, each mouse was given a probe test in which the platform was removed from the pool and the mouse was allowed to swim freely for 1 min. The crossings of target platform were recorded for each mouse. After completing the WMM, mice were kept in a plastic holding cage placed on an electric heater.

### ^1^H-MRS Assessment

^1^H-MRS was conducted on a 7.0T MRI scanner (Bruker PharmaScan 70/16, Bruker, Germany) coupled with a 12-cm-diameter self-shielded gradient system and a 23-mm surface coil to receive signals. Three groups of mice all underwent MRI examination at 6 weeks after transplantation. Animals were anesthetized with 5% isoflurane for induction and 2% isoflurane for maintenance. Animals were fixed on the experimental bed in prone position with the frontal tooth hooked with a tooth bar. During examination, the body temperature was kept at 36–37°C and respiratory frequency was monitored using an animal vital sign guarding system. T2-weighted images were acquired using rapid acquisition relaxation enhancement (RARE) as following parameters: TR = 4000 ms, TE = 24 ms, slice thickness = 0.8 mm, field of view (FOV) = 20 mm × 20 mm, matrix = 256 × 256, and RARE factor = 4. The obtained images were used for guiding subsequent locations of MRS examination.

**Figure 1:**
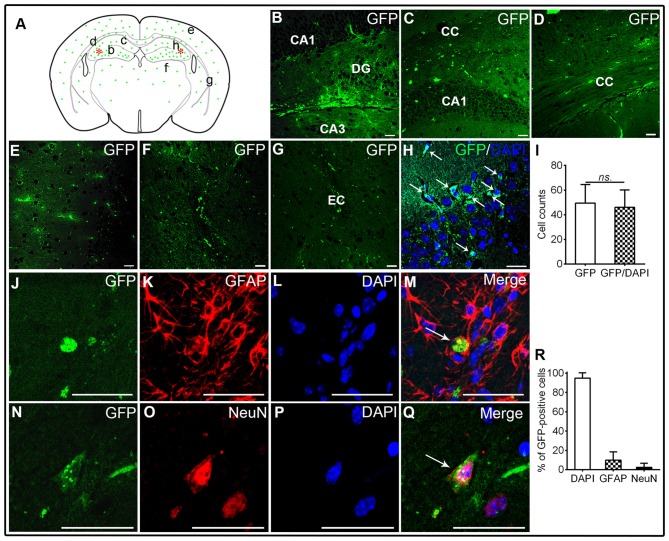
**Transplantation of human brain-derived neural stem cells (hNSCs) into hippocampal CA1 region of amyloid precursor protein (APP)/presenilin 1 (PS1; Alzheimer’s disease, AD) mice. (A)** Schematic drawing of a typical coronal brain section, illustrating the distribution of engrafted green fluorescent protein (GFP)-labeled cells (green dots). Asterisks represent the transplantation sites. Lowercase letters indicate sites where corresponding images **(B–G)** were taken. **(B,C)** Transplanted hNSCs engrafted into the hippocampus, and they migrated extensively into corpus callosum **(D)**, frontal cortex **(E)**, thalamus **(F)** and external capsule **(G)**. **(H,I)** GFP-labeled hNSCs co-localized well with 4′,6-diamidino-2-phenylindole (DAPI; white arrow). Some of hNSCs differentiated into GFAP-astrocytes or mature neurons **(J–R)**. There was no significant difference in counts between GFP-labeled and GFP/DAPI co-labeled cells (*ns.* = *p* > 0.05). Scale bar: 50 μm.

Two regions of interest (ROI) sizing 20 mm^3^ × 20 mm^3^ × 18 mm^3^ were located on the right hippocampus and adjacent cortical region (Figure 1). The ROI location was placed on the coronal, axial and sagittal planes of mice brains. To control the consistency of ROI placements two experienced investigators simultaneously checked the location. MRS data was collected using point-resolved water suppression pulse sequence (PRESS) as following parameters: TR = 2500 s, TE = 20 ms, EF = 1000, *T* = 40 min. The obtained data was processed with the Bruker processing software 2D WIN-NMR (Bruker-Franzen Analytic, Germany). After baseline correction and adjustment, area under the peak of each metabolite was calculated automatically using the NUTS-NMR Utility Transform Software (AcornNMR, Livermore, CA, USA). The evaluated metabolites included NAA, Cho, Glu, mI and Cr. Because Cr is consistent in various diseases, it was used as the internal standard to calculate NAA/Cr, Cho/Cr, Glu/Cr and mI/Cr in this study.

### Histological Examination

For histological examination, mice were re-anesthetized and subject to cardiac perfusion with 10% formalin (PHs7.4, 4°C). The brain was totally removed, segmented into 5-mm-thick coronal slices and then post-fixed for at least 24 h. In particular, the slice involving the middle part of dorsal hippocampus was collected from bregma 0 ventrally to −5.0. The selected slices were dehydrated by gradient ethanol, embedded in paraffin and cut into coronal sections (6 μm). Two series of brain sections were collected from −1.0 to bregma −1.4 and from −2.0 to bregma −2.4 for staining.

Nissl staining was used to evaluate neuronal loss. Based on earlier reports (Fu et al., 2016), sections were hydrated and incubated in 1% cresyl violet acetate at 60°C for 40 min. After three washes with PBS, sections were decolorized with 95% ethanol till appearing clear. Finally, sections were dehydrated, dried and cover-slipped. To reveal Aβ depositions, immunohistochemistry (IHC) was performed based on manufacturer’s instructions. Briefly, hydrated sections were blocked with 10% goat serum, and then incubated with Aβ_1–16_ antibody (6E10, 1:500, Covance/Signet Laboratories, Dedham, MA, USA) for at 4°C overnight. After three washes with PBS, sections were stained with secondary antibody (HRP-labeled anti-rabbit/mouse IgG), visualized with DAB (ZSJQ, Beijing, China), and counterstained with hematoxylin. The stained sections were captured using a Leica digital camera (TCS SP5, Leica, Wetzlar, Germany). Immunofluorescent labeling was conducted based on standard protocols as we described previously. Primary antibodies included glial fibrillary acidic protein (GFAP, 1:400, Millipore, Billerica, MA, USA), Neuronal Nuclei (NeuN, 1:500, Millipore, Billerica, MA, USA), synaptophysin (SYP, 1:200, Sigma, St. Louis, MO, USA), and microtubule associated protein 2 (MAP2, 1:400, Ruiying Biological, Suzhou, China). Primary antibodies were incubated at 4°C overnight and visualized with appropriate Alexa Fluor conjugated secondary antibodies (Invitrogen, Carlsbad, CA, USA). All sections were counterstained with 4′,6-diamidino-2-phenylindole (DAPI, ZSJQ, Beijing, China). Immunofluorescent sections were visualized using a laser scanning confocal microscope (TCS SP5, Leica Microsystems, Heidelberg, Germany).

For quantification analysis, sections were coded and the captured images were analyzed using the ImageJ software package (National Institutes of Health, Bethesda, MD, USA) by two blinded investigators. On Nissl-stained sections, five microscopic fields from the hippocampal CA1, CA3, DG regions and cortical pyramidal (PCL) and multiform cellular layers (MCL) were selected to calculate neuronal counts in each view at 40× magnifications. Whereas Aβ plaque area and intensity was calculated on 6E10-stained sections of the frontal cortex and hippocampus, the positive-staining area was calculated on SYP- and MAP2-stained sections. Large images were scanned on positive sections using the confocal microscope to observe the distribution of engrafted NSCs and calculate the cell counts in multiple brain regions.

### ELISA for Aβ Levels

The Aβ peptides (including soluble and insoluble Aβ1–40 and 1–42) in the cortex and hippocampus were extracted according to previous studies. Briefly, frozen brain tissue were weighed, added with freshly-prepared Trisbuffered saline (TBS; 20 mM Tris-HCl, 150 mM NaCl, PH 7.4) at 4:1 (TBS volume/brain wet weight), and homogenized on a mechanical Fluka homogenizer. The homogenate was centrifuged at 20,000 g for 30 min at 4°C. The supernatant containing soluble Aβ peptides was collected and stored at −80°C. The sediment containing insoluble Aβ peptide was rehomogenized with an equal volume of TBS plus 5 M guanidine HCl (pH 8.0) and incubated at room temperature. After re-centrifugation, the supernatant was collected and stored at −80°C. Aβ1–40 and Aβ1–42 levels were quantified by ELISA kit according to manufacturer’s instructions (Invitrogen, Carlsbad, CA, USA).

### Ultrastructural Analysis

Transmission electron microscopy (TEM) was performed to observe the ultrastructural changes. Tissue samples were prepared as we described previously (Li et al., 2010). In brief, mice were re-anesthetized and perfused cardically with 0.9% sodium chlorine (4°C) followed by 2.5% glutaraldehyde in 0.01M PBS (pH 7.4, 4°C). The brains were cut into 1-mm coronal slices, the hippocampus and frontal lobe were trimmed into blocks of approximately 2 mm^3^ × 1 mm^3^ × 0.5 mm^3^ (length × width × thickness) and then post-fixed at 4°C until processing. The tissues were rinsed in double-distilled water, dehydrated in gradient ethanol and propylene oxide. Finally, the blocks were embedded in epoxy resin, cut into 80-nm sections, and stained with uranyl acetate and bismuth subnitrite to increase contrast. The ultrathin sections were examined under TEM (JEM-1400plus; JEOL Ltd, Japan).

### Statistical Analysis

All results were expressed as means ± SEM. Statistical analyses were performed using GraphPad Prism 6.0 (GraphPad Software, Inc., La Jolla, CA, USA). All continuous variables were tested to confirm that they fit a normal distribution before further analysis. Difference comparisons among three groups were analyzed with one-way ANOVA, followed by *post hoc* Tukey’s test (*α* = 0.05) to assess the difference between any two groups. A repeated measures ANOVA was used to analyze the MWM learning curve. Difference between two groups was compared using unpaired *t*-test. Difference was considered as significant when *p* < 0.05.

## Results

All mice completed the experiment and there was no death associated with transplantation of hNSCs or PBS. No additional adverse signs were observed in mice receiving hNSC implantation relative to those with PBS treatment.

### Survival, Migration and Differentiation of Transplanted hNSCs in APP/PS1 Transgenic Mice of AD

The GFP-labeled hNSCs were used in this study to identify the location of engrafted cells in various brain regions of APP/PS1 mice at 6 weeks after transplantation. The GFP-positive cells were widely distributed from transplantation sites (hippocampal CA1 region) to ambient regions including the corpus callosum, adjacent cortex, and even to distant regions like thalamus and external capsule (Figures 1A–G). These cells were well co-localized with DAPI staining, confirming the nature of transplanted NSCs (*t*-test; *t*_(1,78)_ = 1.05, *p* = 0.38; Figures 1H,I). To disclose the phenotype of engrafted hNSCs, glial cells and neurons were labeled by the astrocyte (GFAP) or neuronal marker (NeuN). Whereas a minority of GFP-labeled hNSCs expressed GFAP in the corpus callosum, external capsule and thalamus (Figures 1J–M), some others were co-localized with NeuN mainly in the hippocampus and cortex (Figures 1N–Q). Quantification analysis showed that most of GFP-labeled cells co-localized well with DAPI staining (94.7 ± 5.6%), wherein some cells differentiated into GFAP-astrocytes (9.8 ± 8.7%) or mature neurons (2.4 ± 4.4%; Figure 1R). These results indicate that hNSCs survived, engrafted, migrated, differentiated and matured into neural cell types of central nervous system (CNS) after transplantation in AD mice brains.

### Alleviated Cognitive, Learning and Memory but Not Anxiety Deficits by hNSC Transplantation in AD Mice

**Figure 2:**
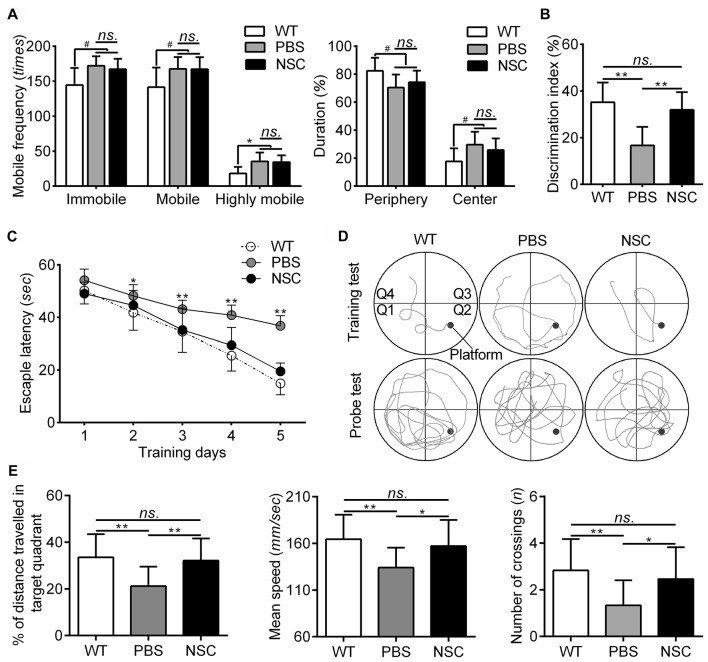
**hNSCs rescued recognition, learning and memory function but not anxiety level in APP/PS1 mice. (A)** Analysis of open field test (OFT) tests. There was no significant difference in frequencies of mobile, immobile and highly mobile states, as well as % of duration in periphery and central zones between the PBS and NSC group (*ns.* = *p* > 0.05), ^#^*p* < 0.05 vs. wild-type (WT) mice. **(B)** There was no difference in discrimination index (DI) between the WT and NSC group (*ns.* = *p* > 0.05), **p < 0.01 vs. the PBS group.** (C)** Escape latencies in five training days of the morris water maze (MWM). Significant interactions were found for the latency from training day 2 (*p < 0.05; **p < 0.01), where NSC group mice decreased the latency to the hidden platform. No significant difference in latencies was found between the NSC and WT group (*ns.* = *p* > 0.05). **(D)** Representative training (5th day) and probe traces of mice in three groups. **(E)** Analysis of the probe test. There was no significant difference in % of distance traveled in target quadrant, mean speed and number of crossings between the WT and NSC group (*ns.* = *p* > 0.05), *p < 0.05; **p < 0.01 vs. the PBS group.

The behavioral changes were assessed in all mice before histological examination, the results of which are summarized in Figure 2. The OFT was used to assess the anxiety of AD mice. In the test, both the PBS and NSC group mice exhibited higher frequencies of immobile, mobile and highly mobile states and spent more time in the central zone and lesser time in the periphery zone than WT mice (ANOVA with *post hoc* Tukey’s test, all *p* < 0.05; Figure 2A), suggesting that AD mice were more hyperactive and anxious. However, no significant difference was observed between two groups of AD mice in any of the five parameters including mobile frequencies and time spent in each zone (all *p* > 0.05; Figure 2A). Thus, hNSCs did not significantly alter anxiety level in AD mice. In the NOR task, NSC group mice showed notably higher DI values than PBS group, but there were no significant difference in DI between NSC group and WT mice (ANOVA with *post hoc* Tukey’s test, *p* = 0.001 and *p* = 0.60 respectively; Figure 2B), suggesting that hNSCs restored the impaired recognition in AD mice.

The MWM test was used to evaluate the learning and memory of AD mice after transplantation. In the whole test, NSC group mice showed similar performance with WT mice. In training tests, WT mice showed significantly decreased escape latency from day 2 compared with PBS group (repeated measures ANOVA with *post hoc* Tukey’s test, *p* < 0.05 for day 2; *p* < 0.01 for day 3–5). The NSC group mice showed significantly decreased escape latency from day 3 compared with PBS group (*p* < 0.01 for day 3–5; Figures 2C,D). In the probe test, % of distance traveled in target quadrant, mean speed, as well as crossings of target platform was significantly reduced in the PBS group mice as compared with WT mice (ANOVA with* post hoc* Tukey’s test, all *p* < 0.05; Figures 2D,E). These parameters were significantly increased in the NSC group mice (all *p* < 0.05 vs. the PBS group) and there was no difference between the NSC and WT group (all *p* > 0.05), suggesting that the learning and memory deficits were significantly alleviated in AD mice after hNSC transplantation. These results denote that hNSCs transplanted in the hippocampus rescued learning and memory deficits in AD mice.

### Reduced Soluble Aβs but Not Insoluble Aβs and Plaque Burden by hNSC Transplantation in AD Mice Brains

**Figure 3:**
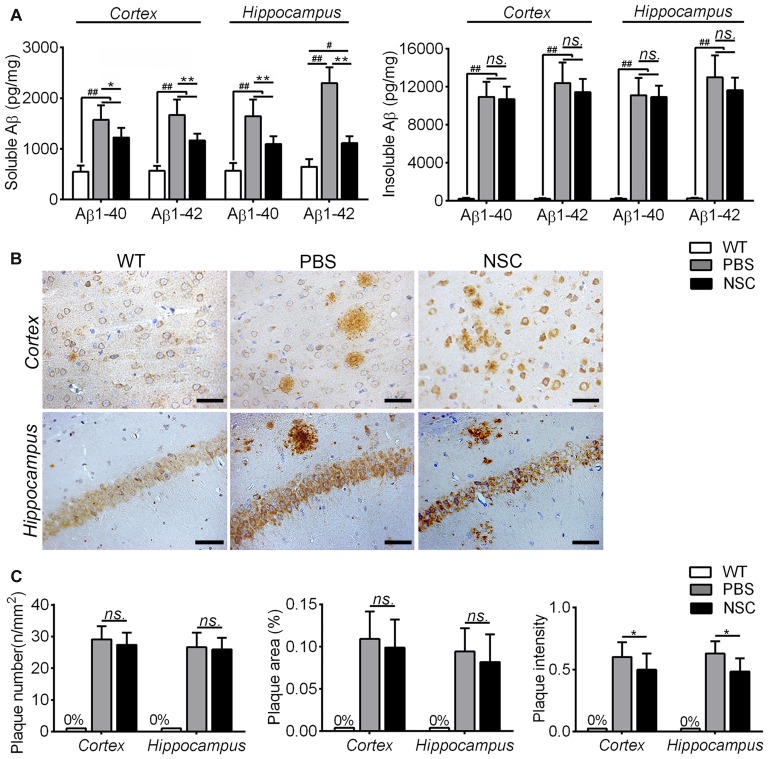
**hNSCs slightly alleviated Aβ pathology in APP/PS1 mice. (A)** Analysis of ELISA results. The soluble and insoluble Aβs were significantly higher in the frontal cortex and hippocampus of the PBS and NSC group than that of WT mice (^#^*p* < 0.05; ^##^*p* < 0.01 vs. WT mice). Though soluble Aβs was significantly reduced in the NSC group (*p < 0.05; **p < 0.01 vs. the PBS group), no significant difference was found in insoluble Aβs between the NSC and PBS group (*ns.* = *p* > 0.05). **(B)** Representative 6E10-staining images in the cortex and hippocampus of PBS, NSC group and WT mice. Scale bar: 20 μm. **(C)** There were no significant difference in the Aβ plaque number and area in the frontal cortex and hippocampus between the PBS and NSC group (*ns.* = *p* > 0.05). However, the plaque intensity was significantly reduced in the NSC group (*p < 0.05, vs. the PBS group).

To assess effects of hNSCs on Aβ pathology in AD brain, the Aβ level and plaque burden were both assessed in the NSC, PBS and WT group mice. Because insoluble Aβs were intermediates from soluble Aβs to plaques, two Aβ forms were tested separately, and the results are summarized in Figure 3A. Compared with WT mice, soluble and insoluble Aβ1–40 and Aβ1–42 peptides were all significantly increased in the frontal cortex and hippocampus of PBS group mice (ANOVA with *post hoc* Tukey’s test, all *p* < 0.05). Compared with PBS group, soluble Aβ1–40 and Aβ1–42 were significantly reduced in the frontal cortex and hippocampus of NSC group mice (all *p* < 0.05), to the equivalent levels as exhibited by WT mice. However, insoluble Aβ1–40 and Aβ1–42 were still significantly higher in the cortex and hippocampus of NSC group mice than that of WT mice (all *p* < 0.05), and there was no significant difference between the PBS and NSC group mice (all *p* > 0.05). These results suggest that hNSCs significantly reduced soluble but not insoluble Aβ1–40 and Aβ1–42 levels in the cortex and hippocampus of AD mice.

The Aβ plaque burden was evaluated on 6E10-stained sections (Figure 3B). No Aβ-positive staining was observed in the frontal cortex and hippocampus of WT mice. Extensive Aβ plaques were observed in the brains of PBS group mice, and Aβ plaques were alleviated in the NSC group mice compared with PBS group. Quantification analysis showed that the Aβ plaque number, area and intensity was both reduced in the cortex and hippocampus of NSC group mice compared that of PBS group, but the reduced plaque number and area did not reach significance (*t*-test, all *p* > 0.05; Figure 3C). These results denote that hNSCs only slightly alleviated the Aβ plaque burden in the brains of AD mice.

### Rescued Neuronal Loss and Connectivity by hNSC Transplantation in AD Mice Brains

**Figure 4:**
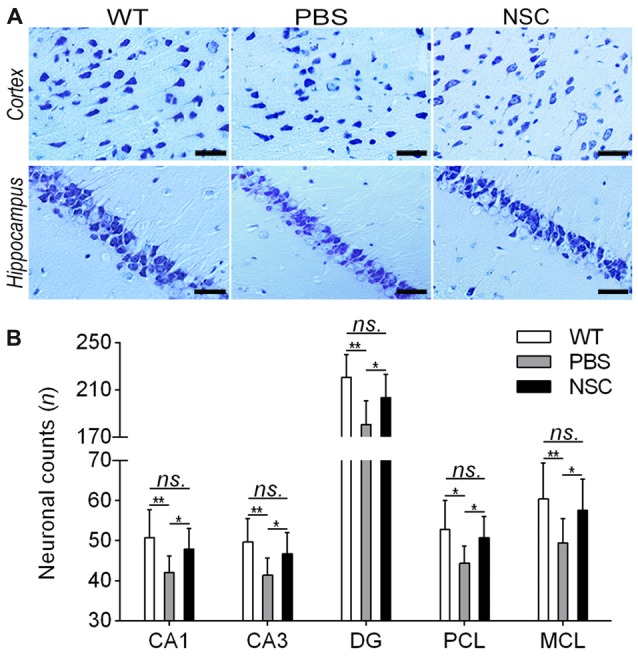
**hNSCs rescued neuronal loss in APP/PS1 mice. (A)** Nissl-staining images in the frontal cortex and hippocampus of PBS, NSC group and WT mice. Scale bar: 20 μm. **(B)** Comparisons of neuronal counts per view (40×) in the hippocampal CA1, CA3, DG regions and cortical pyramidal cellular layer (PCL) and multiform cellular layers (MCL) areas among three groups of mice. There was no significant difference in neuronal counts between the NSC group and WT mice (*ns.* = *p* > 0.05), *p < 0.05; **p < 0.01 vs. the PBS group.

Nissl staining was conducted to assess effects of hNSCs on neuronal loss in AD brain; representative images are shown in Figure 4. Compared with WT mice, PBS group mice showed typical Alzheimer’s pathology, including neuron loss, nucleus shrinkage or disappearance of Nissl bodies in the frontal cortex and hippocampus (Figure 4A). Compared with PBS group, the neuronal number was significantly increased and the cell organization was notably improved in the hippocampal CA1, CA3, DG regions and cortical PCL and MCL areas of NSC group mice (ANOVA with *post hoc* Tukey’s test, all *p* < 0.05; Figure 4B). However, no notable difference was observed in neuronal counts between NSC and WT group mice (all *p* > 0.05). These results imply that hNSCs improved the number and structure of neurons in AD mice brains.

**Figure 5:**
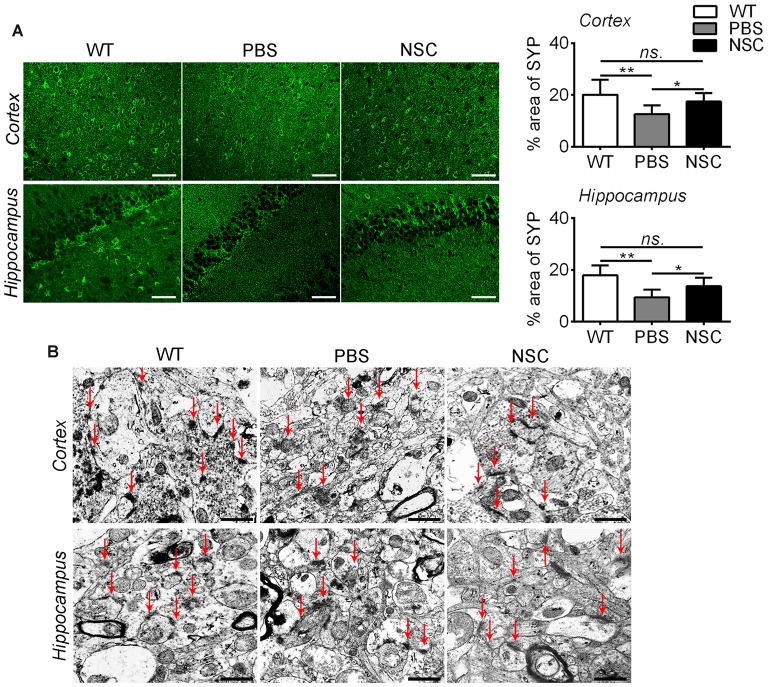
**hNSCs rescued synapses in APP/PS1 mice brains. (A)** SYP-staining sections in the brains of PBS, NSC group and WT mice, and quantification of SYP-staining in the frontal cortex and hippocampus of three groups of mice. There was no significant difference in % area of SYP between the NSC group and WT mice (*ns.* = *p* > 0.05), *p < 0.05; **p < 0.01 vs. the PBS group. Scale bar: 20 μm. **(B)** Ultrastructure analysis showed that synapses (red arrow) were dense and intact in the cortex and hippocampus of WT mice, appeared structurally loose and sparsely distributed in PBS group mice, but regained well-structured shapes and densely spaced in NSC group mice. Arrow indicates synapses. Scale bar: 1 μm.

Synaptic injury is another feature of AD. To assess how synapses changed after hNSC transplantation, we examined the synaptophysin immunoreactivity. As shown in Figure 5A, synaptic density was significantly reduced in the frontal cortex and hippocampus of PBS group mice compared with WT mice (ANOVA with *post hoc* Tukey’s test, *p* = 0.002 and *p* = 0.001 respectively). HNSCs significantly increased synaptic density in AD mice compared with PBS treatment (*p* = 0.034 and *p* = 0.013 respectively), to levels equal to WT mice (*p* = 0.331 and *p* = 0.130 respectively). Ultrastructural analysis showed that synapses were sparsely distributed and structurally loose in the frontal cortex and hippocampus of PBS group mice, whereas most synapses were densely spaced and maintained a well-structured shape in NSC group mice (Figure 5B).

**Figure 6:**
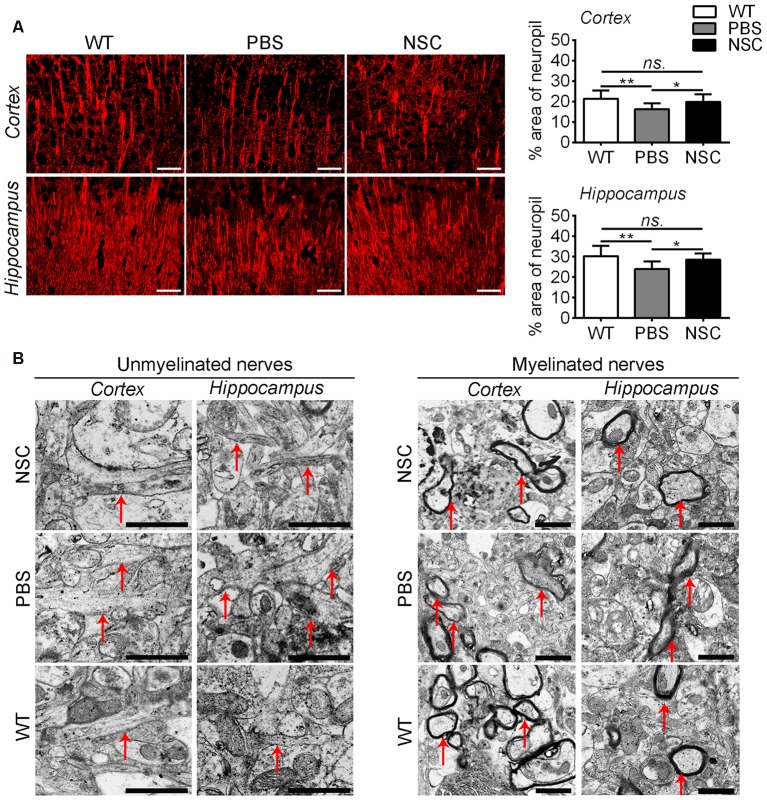
**hNSCs improved nerve fibers in APP/PS1 mice brains. (A)** Microtubule associated protein 2 (MAP2)-staining sections in the brains of PBS, NSC group and WT mice, and quantification of MAP2-staining in the frontal cortex and hippocampus of three groups of mice. There was no significant difference in % area of neuropil between the NSC group and WT mice (*ns.* = *p* > 0.05), *p < 0.05; **p < 0.01 vs. the PBS group. Scale bar: 20 μm. **(B)** Ultrastructure analysis showed the enlargement and disorganization of unmyelinated nerves in the cortex and hippocampus of PBS group mice; the damage was alleviated in NSC group. Likewise, myelinated nerves maintained relatively intact in myelin sheath in NSC group and WT mice. Arrow indicates the nerve fibers. Scale bar: 1 μm.

Synaptic injury is often associated with damage of neuronal dendrites in AD brain. Thus, brain sections were labeled with a MAP2 antibody to assess synapto-dendritic damage (Figure 6). Results showed that NSC and WT group mice exhibited comparable levels of MAP2 immunoreactivity in the frontal cortex and hippocampus (ANOVA with *post hoc* Tukey’s test, *p* = 0.316 and* p* = 0.321 respectively; Figure 6A). Compared with PBS group, NSC group mice exhibited a notable increase in % area of the neuropil covered by MAP2-staining dendrites (*p* = 0.019;* p* = 0.010), and there was no significant difference in MAP2 immunoreactivity between NSC group and WT mice (*p* = 0.316 and *p* = 0.321 respectively). Ultrastructure analysis showed that unmyelinated nerves appeared enlarged in appearance and mussy in organization in the frontal cortex and hippocampus of PBS group mice, whereas the damage was notably repaired in NSC group mice. Likewise, compared with PBS group, myelinated nerves were relatively intact in myelin sheath in both NSC group and WT mice (Figure 6B).

### Improved Neuronal Metabolic Activity by hNSC Transplantation in AD Mice Brains

**Figure 7:**
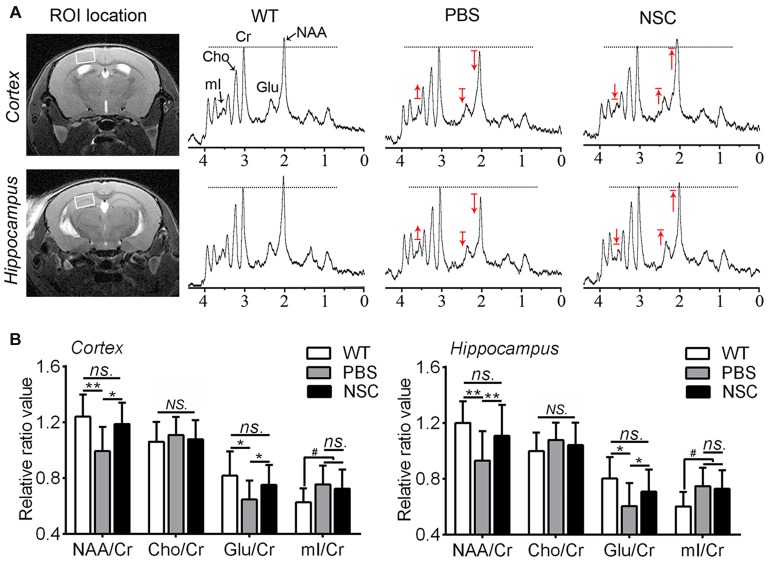
**hNSCs improved brain metabolic activity in APP/PS1 mice. (A)** Regions of interest (ROI) location in the brain (white box) and representative spectra from NSC, PBS group and WT mice. N-acetylaspartate (NAA) and Glu peaks are lowered while myo-inositol (mI) is elevated in the frontal cortex and hippocampus of PBS group mice compared with WT group; hNSCs increased NAA, Glu but decreased mI in AD mice compared with PBS treatment. The red arrow indicates direction of metabolic changes whereas line represents the control level of each metabolite. **(B)** Quantification of the relative ratios of each metabolite to creatine (Cr) in the cortex and hippocampus of three groups of mice. There was no significant difference in NAA/Cr and Glu/Cr between the NSC group and WT mice and no difference in mI/Cr between the NSC and PBS group (*ns.* = *p* > 0.05), ^#^*p* < 0.05 vs. WT mice; *p < 0.05, **p < 0.01 vs. the PBS group. No significant difference in Cho/Cr was found among three groups of mice (*NS.* = *p* > 0.05).

^1^H-MRS was used to assess brain metabolic changes after transplantation in AD mice, the results of which are summarized in Figure 7. Compared with WT mice, NAA and Glu peaks decreased while Cho and mI peaks increased in the frontal cortex and hippocampus of PBS group mice (Figure 7A). Compared with PBS group, NAA and Glu peaks were elevated while Cho and mI was lowered in brains of NSC group mice, to levels exhibited by WT mice. Quantification analysis showed that NAA/Cr and Glu/Cr were significantly higher in the frontal cortex and hippocampus of NSC group mice than that of PBS group (ANOVA with *post hoc* Tukey’s test, all *p* < 0.05), and there was no significant difference between NSC group and WT mice (all *p* > 0.05; Figure 7B). Likewise, mI/Cr was significantly higher in the cortex and hippocampus of PBS group mice than that of WT mice (*p* = 0.043 and *p* = 0.023 respectively). But, no significant difference was found in mI/Cr between NSC and PBS group mice (*p* = 0.829 and *p* = 0.776 respectively), indicating that increased mI was not significantly reduced by hNSCs. By contrast, there was no notable distinction in Cho/Cr among three groups of mice (ANOVA, *F*_(2,33)_ = 0.395, *p* = 0.677; *F*_(2,33)_ = 1.01, *p* = 0.374, respectively). These results indicated that hNSCs significantly elevated the NAA and Glu level but hardly affects the mI and Cho level in AD mice brains, suggesting that hNSC transplantation improved neuronal metabolic activity in AD brains.

## Discussion

In this study, we examined the effects of hNSCs transplanted in the hippocampus on recognition ability by NOR task, hippocampus-dependent learning and memory by MWM test, Aβ pathology and neural degeneration by histological examination, as well as brain metabolic level by MRS. Our data showed that the recognition, learning and memory ability but not anxiety level was rescued by hNSC transplantation in AD mice. Histological examination indicated that implanted hNSCs survived, engrafted and differentiated in AD mice brains, that neuronal, and synaptic and nerve density was significantly increased after hNSC transplantation, and that synapses and nerve fibers maintained relatively well-shaped structures in the brains of NSC-treated mice. *In vivo* MRS evaluation denoted that brain metabolic activity was significantly improved after hNSC transplantation in AD mice. But interestingly, implanted hNSCs did not notably reduce insoluble Aβs and Aβ plaque burden in AD mice brains. Our findings suggest that it is feasible to transplant hNSCs in AD mice, providing preclinical evidence that hNSC transplantation can be a hoping choice for patients with AD.

The transplantation of human-derived NSCs in experimental animals is an essential stage to future usage of hNSCs in patients with AD. Recently, emerging studies reported transplantation of hNSCs in mice with various disease models (Lee I.-S. et al., 2015; Zuo et al., 2015; Haus et al., 2016). While some studies reported the differentiation failure of engrafted hNSCs in mice (Lee S.-R. et al., 2015), more studies showed the predominant differentiation of hNSCs into astrocytes (Gao et al., 2006; Lee I.-S. et al., 2015). The different differentiation patterns could be associated with the source of human cells, cell preparation, as well as difference in animal models. Our studies showed that transplanted NSCs well survived, extensively migrated and engrafted into broad brain regions and that most of engrafted hNSCs differentiated majorly into astrocytes and occasionally into neurons. Whereas the astrocytic differentiation mainly occurs in the corpus callosum and thalamus, the neuronal destination mainly locates in the cortex and hippocampus. Because neuronal replacement by grafted cells is not possible, the beneficial effects of hNSCs observed in this AD model are through cell complement rather than replacement. Furthermore, we find no adverse effects in NSC-treated mice compared with PBS treatment and both groups of mice exhibited no mortality after transplantation, supporting the safety and feasibility of using hNSCs.

Cell rejection is an ongoing issue challenging the NSC transplantation therapy especially for using xenogeneic transplants (Lee I.-S. et al., 2015). Due to difference in cell sources and transplantation sites, the survival and differentiation rates of implanted xenogeneic NSCs are differently reported across studies (Ager et al., 2015; Haus et al., 2016). Some studies reported the injection of immunosuppressive drugs in combination with cell transplantation to enhance the survival of xenogeneic NSCs (Ager et al., 2015; Lee I.-S. et al., 2015). In this study, however, we injected hNSCs into the hippocampus of AD mice without addition of immunosuppressive agents. Encouragingly, we found a favorable survival, migration and differentiation of transplanted hNSCs in the brain of AD mice. This may be related with the fact that hippocampus where we implanted hNSCs is one region related with neurogenesis and can provide appropriate microenvironment for engrafted NSCs (Vadodaria and Gage, 2014; Li et al., 2015). Moreover, the procedure for isolating hNSCs has been optimized in our experiments and transplanted hNSCs have been shown to survive well in the brain of Parkinson’s mice (Zuo et al., 2015). Thus, this study suggests that the well-isolated hNSCs can be used for transplantation in AD brains, at least in animal models. Although still scant, this study provides new hope for future use of hNSCs in the treatment of AD. In particular, advances in induced pluripotent stem cell technology make it possible to generate sufficient homogeneous stem cells that can be used for cell therapy (Takamatsu et al., 2014; Pen and Jensen, 2016), which will certainly facilitate the transplantation of hNSCs in AD.

APP/PS1 mice have been widely used to mimic behavioral changes associated with AD (Li X. et al., 2016). Our earlier studies reported that those mice exhibited notable learning and memory deficits at 8 months of age (Li X.-F. et al., 2016). In this study, we further showed that PBS-treated AD mice exhibited notable disorders in the anxiety and recognition compared with WT mice. By contrast, in AD mice treated with hNSC transplantation, the recognition, learning and memory ability was significantly improved, approximating to the level exhibited by WT mice. However, transplanted hNSCs did not alter the anxiety level of AD mice. Our results are consistent with early studies showing cognitive improvement after NSC transplantation in AD mice (Ager et al., 2015); however, the finding that hNSCs has no effect on anxiety in AD mice has not been reported previously.

APP/PS1 mice exhibited marked increase of Aβ accumulation, consistent with the presentation in patients with AD, especially for those with early- to mid-stage AD (Li X. et al., 2016). Since this model doses not generate tau pathology, it is often used to test therapeutic intervention on Aβ-related pathologies. Although murine NSCs have been shown to improve Alzheimer’s cognition and reduce Aβ load (Blurton-Jones et al., 2009; Chen et al., 2012), the effects of hNSCs on AD are still unknown. In this study, we showed that hNSCs notably reduced soluble Aβs but not insoluble Aβs and plaque area, suggesting that hNSCs may improve Alzheimer’s cognition through other mechanisms rather than only modification of Aβ pathology development. Such a finding is consistent with studies showing that a number of new therapies targeted at Aβ formation failed clinical trials (Selkoe and Hardy, 2016).

Besides Aβ pathology, AD is associated with widespread neuronal loss in brains (Zou et al., 2015). Thus, we calculated the neurons in the frontal cortex and hippocampus of AD mice, finding that neuronal number was significantly increased after hNSC transplantation. Moreover, synaptic loss was shown to be more related with AD-associated cognitive deficits (Shankar et al., 2008; Lo et al., 2013), whereas synaptophysin increase was reported to correlate with cognitive recovery in model mice of AD (Ager et al., 2015). In this study, we found a significant increase of synaptic proteins in the brains of mice receiving hNSC implantation, corresponding to the improved cognition after transplantation. In addition, MAP2 levels were also notably enhanced after hNSC transplantation in AD mice brains, suggesting of increased dendritic density. While decreased dendrites were observed in AD brains, increased dendrites were to reported correlate with cognitive improvement (Rockenstein et al., 2015). Ultrastructure analysis confirmed that hNSCs noticeably improved the structure of synapses and nerve fibers in AD brains. Because nerve fibers and synapses are major constitutors of the neuronal network, increased nerves and synapses will certainly enhance the neuronal connectivity. Thus, we suppose that hNSCs rescue Alzheimer’s cognition by repairing neuronal connectivity through improving the synapse and cytoskeleton. This point is in agreement with the view by Lu et al. (2003), who postulated that NSC transplantation repairs brain tissue by establishing functional neural circuits.

Brain metabolism was assessed using MRS in AD mice. NAA has always been regarded as a specific marker of neurons, which is primarily expressed in neurons within CNS (Ackl et al., 2005). Studies of AD using MRS have shown that reduced NAA was notably restored in patients with AD after medical treatment and that NAA changes could reflect therapeutic effects (Arturo et al., 2004; Jessen et al., 2006). Glu is a major neurotransmitter within mammal CNS that regulates learning, memory and movement, and is closely related with the survival of neurons and plasticity of synapses (Alvarez and Ruarte, 2004). In this study, we found that reduced NAA and Glu were significantly increased in the frontal cortex and hippocampus of AD mice with hNSC transplantation, suggesting that hNSCs increased the neuronal metabolic activity in AD brains. The increased neuronal activity is echoed by enhanced neuronal number in NSC-treated mice brains as revealed by Nissl-staining, associated with cognitive improvement after hNSC transplantation. These results suggest that hNSCs significantly increased neuronal metabolic activity in AD brains that ultimately ends in improvement of Alzheimer’s cognition.

Cho is involved in the formation of cell membrane and nerve myelin, which can reflect the stability of cell membrane. mI is associated with the activation or proliferation of astrocytes, often used as a featured marker of glial cells (Oz et al., 2014). While Cho was shown to increase due to disrupted cell membrane in AD brains, mI was to be elevated because of reactive astrocyte proliferation (Zhang N. et al., 2014). In this study, we showed that Cho and mI were both increased in the frontal cortex and hippocampus of AD mice but only increased mI reached significance. However, we found no notable changes in Cho and mI in mice receiving hNSC transplantation compared with those with PBS treatment. These results imply that hNSCs had less effect on neural cell membrane stability and astrocyte activation in AD brains. Our findings are to some extent disagreeing with some other reports. For example, Chen et al. (2012) demonstrated that transplanted NSCs notably decreased mI in the hippocampus of APP/PS1 mice. This may be related with the fact that the used NSCs were from rodents but not human, and the tested mice was aged at 12 months that were older than ours when glial proliferation was more remarkable. However, due to unclear mechanism of implanted NSCs affecting glial activation, there are no reports accounting for the disputable changes in mI after implantation of different NSC types.

There exist some limitations with this study. First, to reduce the number and sufferings of mice, one additional group of APP/PS1 mice receiving no treatment was not included in the study. Thus, a strict quality control on the study performance and data analysis in each experiment was performed by two investigators blinded to study design to minimize possible variations. Second, this study showed a one-time-point but not longitudinal behavioral and pathological changes after transplantation. This is because earlier studies have shown that NSCs showed the best performance at 6 weeks after transplantation but a systematic evaluation of the therapeutic effects have rarely been performed. Last, this study verified that hNSCs improved Alzheimer’s cognition by enhancing neuronal connectivity and metabolic activity through a cell compensation mechanism. However, the molecular mechanism of neural alleviation after hNSC transplantation is not investigated and further researches are required in future.

In summary, this study shows that hNSC transplantation rescued Alzheimer’s cognition in APP/PS1 Tg mice models of AD. The transplanted hNSCs survived, migrated and differentiated into neural cell types of CNS. Engrafted hNSCs improved the density and structure of neurons, synapses and nerve fibers, enhanced the neuronal metabolic activity, but showed no effects on Aβ plaque burden. Thus, it should that transplanted hNSCs rescue Alzheimer’s cognition by enhancing neuronal connectivity and metabolic activity via a complement mechanism in AD mice brains. This study provides preclinical evidence that hNSC transplantation can be used for treating AD. Further studies are required to explore the molecular mechanism of hNSCs acting in AD brains before being applied to patients with AD.

## Author Contributions

RW and XB designed the research. XL, XS, FZ, JL and ZW performed the research. XL, HZ and XB analyzed the data. XL and RW wrote the article.

## Conflict of Interest Statement

The authors declare that the research was conducted in the absence of any commercial or financial relationships that could be construed as a potential conflict of interest.
